# Uncovering potential autotrophs in coal seams: characterization, enrichment, and genome-resolved metabolisms of a subsurface coal seam microbiome

**DOI:** 10.1128/aem.00624-26

**Published:** 2026-09-24

**Authors:** Djuna Gulliver, Daniel E. Ross, Samuel Flett

**Affiliations:** 1 National Energy Technology Laboratory, Pittsburgh, Pennsylvania, USA; 2 NETL Support Contractor, Pittsburgh, Pennsylvania, USA; Colorado School of Mines, Golden, Colorado, USA

**Keywords:** gas fermentation, coal seam microbiome, carbon conversion

## Abstract

Biological transformation of inorganic carbon into organic compounds is an extremely important biosynthetic process, and many organisms catalyze this reaction autotrophically, using CO_2_ as the sole carbon source. Natural autotrophic pathways are diverse, as are the organisms that employ them, driven in part by the varied ecological niches they inhabit. One understudied ecological niche, coal seams, contains autotrophic acetogens that catalyze an intermediate step in the conversion of coal to methane. Here, we characterize the geochemistry and microbiology of coal seam produced water (CSPW) from the Appalachian Basin and, through continued anaerobic enrichment on H_2_:CO_2_, show enhanced growth and acetate production. Specifically, the enriched CSPW microbiome was comprised of *Proteiniphilum, Proteiniclasticum, Gudongella, Acetobacterium, Propionicimonas,* and *Mesobacillus*. Genome-resolved metabolisms revealed six potential autotrophic pathways, including four natural pathways (the reductive pentose phosphate pathway, the reversed oxidative tricarboxylic acid cycle, the reductive acetyl-CoA pathway, and the reductive glycine pathway) and two proposed synthetic pathways (the serine-threonine cycle and the pyruvate formate-lyase threonine cycle), with many metagenome-assembled genomes encoding multiple pathways. Overall, these findings suggest a nuanced and adaptable carbon conversion strategy, shedding light on carbon reactions in coal seams, and the potential applications for biological production of valuable chemicals/fuels using mixed microbial communities.

**IMPORTANCE:**

Coal seams harbor taxonomically diverse microorganisms, specifically acetogens and methanogens, suggesting an untapped potential for autotrophic carbon conversion. Here, we show that an enriched coal seam-produced water microbiome utilized CO_2_ and/or NaHCO_3_ for growth and produced acetate. Genome-resolved metabolisms revealed that most microorganisms encode multiple autotrophic pathways, with the reductive glycine pathway being the most prevalent. This work provides insight into potential microbial strategies for obtaining and transforming carbon in coal seams, and importantly, with proper engineering of operational parameters and reactor design, the enriched microbiome has the potential to be used as a biocatalyst for alternative carbon conversion technologies.

## INTRODUCTION

Carbon fixation is a critical component of the carbon cycle. Most inorganic carbon in the biosphere is converted to organic matter via the reductive pentose phosphate pathway/Calvin-Benson-Bassham (CBB) cycle (1), but many alternative autotrophic pathways have been discovered (2, 3), including the reverse tricarboxylic acid (rTCA) cycle (4, 5), reductive acetyl-CoA pathway (WLP) (6), reductive glycine pathway (rGlyP) (7), dicarboxylate-4-hydroxybutyrate cycle (DC/4HB) (8), 3-hydroxypropionate-4-hydroxybutyrate (3HP/4HB) cycle (9), the 3-hydroxypropionate (3HP) bicycle (10, 11), the serine pathway (12–14), ribulose monophosphate pathway (RuMP) (15), the xylulose monophosphate pathway (XuMP), and the reverse oxidative TCA (roTCA) cycle (16–18). The taxonomic distribution of these pathways is more widespread across the Bacteria and Archaea domains than once thought, revealing previously unknown taxa as potential contributors to carbon conversion in various ecosystems (19).

Coal seams and other energy-related environments represent underutilized microbial resources for CO_2_ conversion and have a potential unaccounted impact on the global carbon cycle. Microorganisms from coal seams have been characterized across the globe (20–27), particularly for their natural ability to convert coal into methane (28–30), which is paramount for enhancing methane production from coal seams as an alternative to burning coal for energy (31). Within this framework, coal intermediates (e.g., long-chain fatty acids) are degraded to hydrogen and carbon dioxide, which is subsequently converted to acetate or methane via acetogenic bacteria or hydrogenotrophic methanogens, respectively (29, 32, 33). While hydrogenotrophic methanogenesis is more energetically favorable at lower CO_2_ and H_2_ concentrations typically found in subsurface coal seams, acetogens may still play an important yet unrecognized role (26).

CO_2_-to-product technologies convert waste carbon into valuable fuels/chemicals from stranded sources, including electrochemical conversion, thermo/chemical conversion (Fischer-Tropsch), and biological conversion (34, 35). Direct reduction of CO_2_ via non-biological routes requires high energy inputs (e.g., elevated temperature and pressure), has low product selectivity, and has a limited product spectrum, often constrained to single-carbon outputs. On the other hand, biological CO_2_ conversion technologies exploit the natural ability of microorganisms to capture and utilize gaseous one-carbon (C1) compounds at atmospheric pressure and near-ambient temperatures to produce longer chain molecules (36, 37). In addition to CO_2_, microorganisms can utilize various gaseous carbon substrates including industrial waste gases (38), synthesis gas (39, 40), and carbon monoxide (41–44). Through proper engineering controls and reactor design, as well as the expression of non-native pathways, CO_2_ can be biologically transformed into higher-value compounds with high rates and titers.

Here, we performed geochemical and microbiological characterization on three coal seam wells from the Appalachian Basin. Continued anaerobic enrichment of coal seam produced water (CSPW) revealed a mixed microbial community capable of growth via carbon fixation with acetate production. Through 16S rRNA gene amplicon sequencing and metagenome sequencing, we identified six main taxa, each encoding various carbon fixation pathways. These pathways have not yet been characterized in the described taxa, expanding our knowledge of microbial strategies for carbon utilization. In some cases, multiple carbon fixation pathways were encoded in the same microorganism, suggesting a more nuanced and adaptable carbon metabolism. This work demonstrates that coal seams represent an underutilized microbial resource for biological CO_2_ conversion technologies and provides a better understanding of their contribution to carbon transformations in subsurface energy systems.

## MATERIALS AND METHODS

### Source of inoculum

The inoculum for enrichment cultures originated from produced water extracted from coal seam methane wells. Produced water samples were obtained from three coal seam methane wells located in southwestern Pennsylvania, USA, named NV57, NV65, and NV73. Water pumped from the wells was immediately collected in sterile 1 L bottles and analyzed for pH, temperature, conductivity, and total dissolved solids (TDS) using a multi-parameter water quality checker (Horiba Instruments, Japan). Produced water samples were placed on ice for transport to the lab.

### Chemical characterization of coal seam produced water

Major and trace cations (Na, Ca, Mg, K, Fe, Sr, Ba, Li, and Mn) were measured at the Pittsburgh Analytical Laboratory (National Energy Technology Laboratory, Pittsburgh, Pennsylvania, USA) following Environmental Protection Agency (EPA) methods 6010C using ICP-OES (Perkin Elmer Optima 7300 DV). Samples were run in duplicate, and average values were recorded (Table S1).

### Sample preparation and metagenome sequencing of original coal seam microbiome

CSPW samples were immediately frozen upon arrival at the lab or filtered through a 0.2 μm filter and immediately frozen. Biomass from frozen filters was processed with a Qiagen DNA/RNA AllPrep Extraction Kit, according to the manufacturer’s instructions. For shotgun metagenome sequencing, DNA libraries were prepared using the Nextera XT DNA Library Preparation Kit according to the manufacturer’s protocol (Illumina, San Diego, CA). Paired-end sequencing reads (2 × 300 bp) were generated on an Illumina MiSeq with the MiSeq v3 Reagent Kit (600 cycles) (Illumina, San Diego, CA). The sequencing effort resulted in 2,327,364,149–2,566,324,748 total bases, 8,107,566–10,907,432 total reads, and 235.28–287.06 mean read length (Table S2). Raw paired-end metagenome reads were uploaded to the kBase web server (45; https://kbase.us).

### Medium composition

For initial enrichments of CSPW, the following four different medium formulations were tested: freshwater medium, phosphate-buffered medium, ATCC 1019 medium, and DSMZ 135 medium. The medium formulations were chosen based on prior use in the growth of autotrophic bacteria.

“FW” medium: A freshwater medium “FW” was prepared according to Marshall et al. (46) and contained the following (per liter): 0.25 g NH_4_Cl, 0.1 g KCl, 0.212 g MgCl_2_, 0.03 g CaCl_2_, 0.6 g NaH_2_PO_4_, 2.5 g NaHCO_3_, 20 mL ATCC Vitamin Supplement, and 20 mL ATCC Trace Mineral Supplement.

Phosphate medium: A phosphate-buffered medium was prepared according to LaBelle et al. (47) and contained the following (per liter): 0.25 g NH_4_Cl, 0.1 g KCl, 0.212 g MgCl_2_, 0.03 g CaCl_2_, 0.6 g NaH_2_PO_4_, 5.24 g KH_2_PO4, 10.71 g K_2_HPO_4_, 2.92 g NaCl, 20 mL ATCC Vitamin Supplement, and 20 mL ATCC Trace Mineral Supplement.

ATCC 1019 medium: 1019 Acetobacterium medium was prepared according to the ATCC medium recipe and included the following (per liter): 1 g NH_4_Cl, 0.4 g KH_2_PO_4_, 0.4 g K_2_HPO_4_, 0.1 g MgSO_4_, 1 g yeast extract, 3 g NaHCO_3_, 0.5 g L-cysteine, 0.5 g Na_2_S, 10 mL ATCC Vitamin Supplement, and 10 mL ATCC Trace Mineral Supplement.

DSMZ 135 medium: A modified version of DSMZ 135 medium was prepared according to Kracke et al. (48). DSMZ 135 medium contained the following (per liter): 1 g NH_4_Cl, 0.33 g KH_2_PO_4_, 0.45 g K_2_HPO_4_, 0.1 g MgSO_4_, 2 g yeast extract, 10 g NaHCO_3_, 0.5 g L-cysteine, 0.5 g Na_2_S, 20 mL ATCC Vitamin Supplement, and 20 mL ATCC Trace Mineral Supplement. The following modifications were made: 100 mM MOPS buffer (pH 7) was added, Na-resazurin, Na_2_S, and fructose were omitted, and the concentration of L-cysteine increased from 0.5 g/L to 1 g/L.

### Initial growth and enrichment of a CSPW microbiome

Anaerobic Balch tubes containing 10 mL of medium were inoculated with 1 mL of coal seam formation water. Tubes were gassed with a mixture of H_2_:CO_2_ (80:20) (1 atm) and placed in a rotary shaker/incubator (130 rpm, 30°C) in the dark. Growth was monitored for 20–25 days via absorbance at 600 nm (Fig. S1).

### Temperature, agitation, and yeast extract tests

Experiments were performed as described for the initial growth and enrichment of the CSPW microbiome. Reactions were performed over 7 days and grown at 25, 30, and 40°C, with the addition of 0.5, 1, or 2 g/L yeast extract, with or without agitation (200 rpm). All reactions were performed in triplicate. Next, 10 mL of the sterile medium was placed in 25 mL anaerobic Balch tubes, and the headspace was gassed with H_2_:CO_2_ (80:20) (1 atm). Anaerobic Balch tubes were inoculated with 0.1 mL of an anaerobic culture growing at 30°C on H_2_:CO_2_ (80:20).

### Sodium bicarbonate tests

To test various bicarbonate concentrations, DSMZ 135 medium was prepared with 0, 2.5, 5, or 10 g/L sodium bicarbonate. Incubations were performed in 25 mL anaerobic Balch tubes with 10 mL of the medium. Tubes were inoculated with 0.1 mL of a culture growing anaerobically at 30°C, with agitation (200 rpm) on H_2_:CO_2_ (80:20) (1 atm). Optical density was monitored daily, and at the end of the experiment, the samples were taken for DNA sequencing. To obtain samples for pH and high-pressure liquid chromatography (HPLC) analysis, cultures were filtered with a sterile 0.2-µm EZFlow syringe filter (Foxx PES membrane) (Cole Parmer), and the filtered supernatant was analyzed.

### One liter reactors

Larger reactor tests were performed in 1 L DURAN GLS80 stirred bottle reactors (DWK Life Sciences). Reactors were autoclaved and aseptically filled with 500 mL of sterile modified DSMZ 135 medium. Reactors were gassed with H_2_:CO_2_ (80:20) for 1 h and subsequently inoculated with 50 mL of an anaerobic culture growing at 30°C in the mid- to late-exponential growth phase. Reactors were operated at 30°C with stirring (400 rpm). At each time point, reactors were gassed with H_2_:CO_2_ (80:20), and 10 mL of liquid sample was extracted for pH, optical density, and organic acids analysis. An aliquot was immediately frozen at −80°C for downstream DNA extraction and sequencing.

### Growth and extracellular metabolite analysis of cultures

Cell density was measured using the optical density at 600 nm absorbance with a Spectronic 200 spectrophotometer (Thermo Scientific). Fatty acids and alcohols from the liquid supernatant were identified and quantified using HPLC. The HPLC (Shimadzu) was operated at 0.55 mL/min, using an Aminex HPX-87H column (Bio-Rad, Hercules, CA, USA), with a column temperature of 40°C. Organic acids were monitored using a refractive index detector (RID) and a photodiode array detector (PDA) at 210 nm. The mobile phase was 5 mM sulfuric acid. Standards were diluted in deionized water or modified DSMZ 135 medium. All samples (biological triplicates) and standards were analyzed in duplicate (technical replicates).

### DNA extraction and 16S rRNA gene sequencing/analysis

Frozen sample aliquots (1.2 mL) were thawed and spun at 13,500 rpm for 5 min. The supernatant was decanted, and 500 µL to 1 mL of the lysis buffer (RLT plus buffer) (Qiagen DNA/RNA AllPrep Kit) was added. Cell pellets were resuspended by vortexing, and a needle/syringe was used to aid in cell lysis. The DNA/RNA AllPrep Kit (Qiagen) was used to extract DNA following the manufacturer’s instructions. DNA concentration was quantified using the Qubit dsDNA High Sensitivity Assay Kit (Life Technologies, Carlsbad, CA, United States).

To generate 16S rRNA gene amplicons, purified DNA was amplified using 16S rRNA gene primers specific to the V4 region (515F/806R) (49). Each sample was pooled in two technical replicates to ensure that enough polymerase chain reaction (PCR) product was generated. After amplification, PCR products were purified using SPRIselect beads (Beckman Coulter, Pasadena, CA), according to the manufacturer’s protocol. A 4 nM amplicon library was generated by pooling and diluting PCR products. The pooled amplicon library was denatured with fresh 0.2 N sodium hydroxide for 5 min. Libraries were further processed according to the manufacturer’s protocol and sequenced on an Illumina MiSeq sequencer using a 300-cycle V2 Nano kit (Illumina, San Diego, CA).

16S rRNA gene sequences were analyzed using QIIME2 version 2021.4 (50). All 16S sequences were imported as EMPSingleEndSequences and subsequently demultiplexed using the demux emp-single command. DADA2 (51) was used for quality control and to filter out any chimeric sequences from the demultiplexed data. The truncation length for these sequences occurred at 250 bases. In order to determine the taxonomic composition of these samples, we used the classify-sklearn command (52) in conjunction with a pre-trained Naive Bayes classifier trained on Silva 132 99% operational taxonomic units (OTUs) (53, 54) from the 515F/806R region to classify the representative sequences from DADA2.

### Metagenome sequencing, read processing, read assembly, and analysis

Shotgun metagenome sequencing was performed with an Illumina MiSeq using a MiSeq Reagent Kit v3 (600-cycle). Genomic DNA was extracted with a DNA/RNA AllPrep Kit (Qiagen), according to the manufacturer’s instructions. DNA concentration was quantified using the Qubit dsDNA High Sensitivity Assay Kit (Life Technologies, Carlsbad, CA, United States). DNA quality was determined with an Agilent bioanalyzer, using the High Sensitivity DNA Assay Kit (Agilent, Santa Clara, CA, United States). DNA libraries were prepared using the Nextera XT DNA Library Preparation Kit according to the manufacturer’s instructions (Illumina, San Diego, CA). Raw paired-end metagenome reads were uploaded to the kBase webserver (45). Raw read quality was assessed with FastQC v0.11.9. Reads were trimmed with Trimmomatic v0.36 (Sliding window size = 4, sliding window minimum quality = 15) (55). Trimmed read pairs were assessed for quality with FastQC and assembled with metaSPAdes v3.15.3 (56), and the resultant contigs were binned using MaxBin2 v2.2.4 (probability threshold = 0.8, marker set = 107 bacterial marker gene set, and minimum contig length = 1,000) (57). Metagenome-assembled genome (MAG) quality was assessed using QUAST (58) and CheckM v1.0.18 (59). Objective taxonomic assignment of each MAG was performed with GTDB-tk v1.7.0 (60–65). MAG abundance was determined by mapping paired-end metagenome reads to individual MAGs using “Map Reads to a Reference Sequence” (V1.0.2, default parameters), which utilizes SAMtools with the Burrows-Wheeler Aligner (BWA) algorithm (66).

### Metagenome-assembled genomes and relevant metabolic pathways

MAGs were uploaded to the RAST server and annotated using the RASTtk pipeline, set to automatically fix errors and backfill gaps (67–69). The highest quality MAGs were analyzed for enzymes involved in all relevant autotrophic pathways. First, pathway enzyme names (including known synonyms) and Enzyme Commission (EC) numbers were cataloged, and the search function in RAST was utilized to explore the presence/absence in each individual MAG. Next, relevant amino acid sequences were searched against the NCBI non-redundant (nr) database using BLASTp, and the top hits were determined for the CBB cycle (Table S5), roTCA cycle (Table S6), WLP (Table S7), reductive glycine pathway (Table S8), and the serine-threonine cycle (Table S9). In cases where all but one or two steps in the pathway were present, amino acid sequences of the missing enzyme found in the genomes of closely related taxa (same genus) were used as BLASTp input for sequence alignment in RAST. For example, a search of the *Proteiniphilum* sp. CSPW MAG for serine deaminase (EC 4.3.1.17) yielded no results. Examination of six sequenced *Proteiniphilum* genomes revealed that all encode serine deaminase (L-serine ammonia lyase), and the amino acid sequence from *Proteiniphilum saccharofermentans* M3/6 was used to query the *Proteiniphilum* sp. CSPW MAG, which had poor alignment to a putative inner membrane protein (27% identity, 43% positive, E-value = 6e-4). The “putative inner membrane protein” amino acid sequence was then queried using the BLASTp (protein-protein BLAST) nr database, which had 99% coverage and 84.78% identity to L-serine ammonia-lyase from a *Proteiniphilum* sp. (accession: WP_323424130.1).

## RESULTS

### Geochemical and microbiological characterization of unenriched coal seam produced water

**Fig 1:**
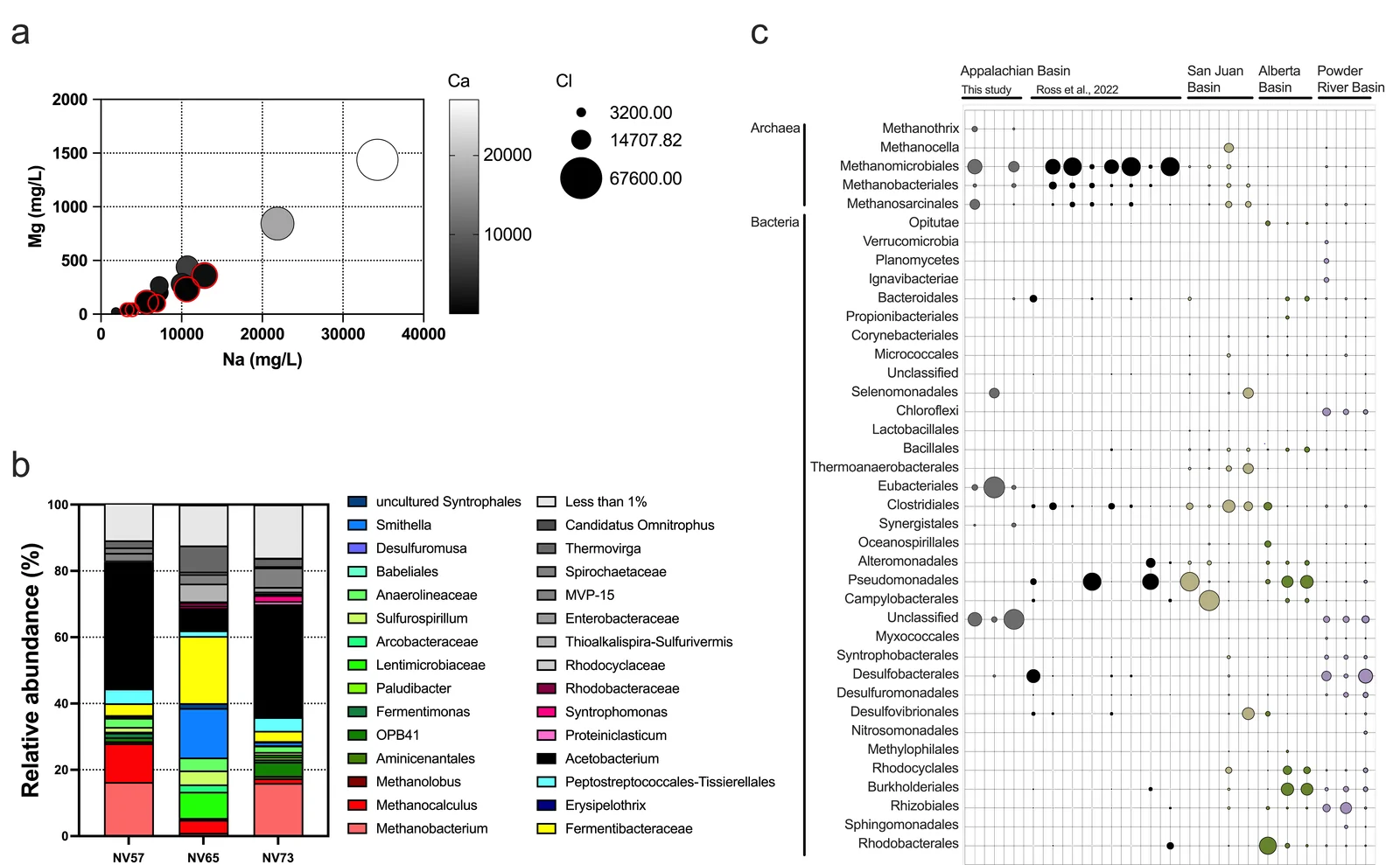
Geochemical and microbiological characterization of CSPW from the Appalachian Basin. (**a**) Major cations and anions of produced water. Data from this study (red outline) were plotted against data from a previous study (25). (**b**) Taxonomic characterization of the original CSPW microbiome with 16S rRNA gene amplicon sequencing. (**c**) Taxonomic characterization of unassembled metagenome reads with comparison to metagenomic samples from other coal basins (adapted from reference 25 with permission of the publisher).

Coal seam produced water from the Appalachian Basin was sampled from multiple wells in southwestern Pennsylvania, USA. Geochemical and microbiological data were named after the coal seam well they originated from (NV57, NV65, and NV73). Fluid chemistry was assessed both onsite and in the lab. TDS ranged from 8.71 g/L to 32.14 g/L, and the pH for all samples throughout the seasons was circumneutral to slightly basic, with a range of 6.58–8.09 (Table S1). The geochemical profiles (major cations/anions) were similar to the measurements of coalbed methane wells previously sampled within the Appalachian Region (Fig. 1a).

To identify the extant microbiome of Appalachian Basin coal seams, 16S rRNA gene amplicon sequencing and metagenome sequencing were performed on samples from wells NV57, NV65, and NV73. Amplicon data revealed taxonomically diverse samples, and microorganisms with the highest relative abundance belonged to *Acetobacterium, Methanocalculus,* Fermentibacteraceae, *Methanobacterium,* and *Smithella* (Fig. 1b). Taxonomic profiling of unassembled metagenomic reads revealed an abundance of Methanomicrobiales and Eubacteriales (Fig. 1c), similar to metagenomes from Appalachian Basin coal seams (25), yet distinct from microbial populations found in coal seams from different geographic regions (e.g., San Juan Basin, Alberta Basin, and Powder River Basin). However, it should be noted that 33% and 70% of metagenome reads were unclassified for samples NV57 and NV73, respectively, suggesting that much of the potential diversity within these samples is novel and/or unaccounted for.

### CSPW microbiome enrichment

To enrich autotrophic microorganisms, such as *Acetobacterium* found in our original sample, unfiltered CSPW was inoculated into four different media with H_2_:CO_2_ (80:20) gas. Samples (named NV57, NV65, and NV73) were incubated at 30°C for over 20 days, and the best growth occurred in bicarbonate-buffered medium (ATCC 1019 and DSMZ 135), while little to no growth was observed with FW minimal medium or phosphate medium (Fig. S1). Specifically, the enrichment from well NV65 reached the highest optical densities and was chosen for further testing (herein, sample NV65 is referred to as the CSPW microbiome).

**Fig 2:**
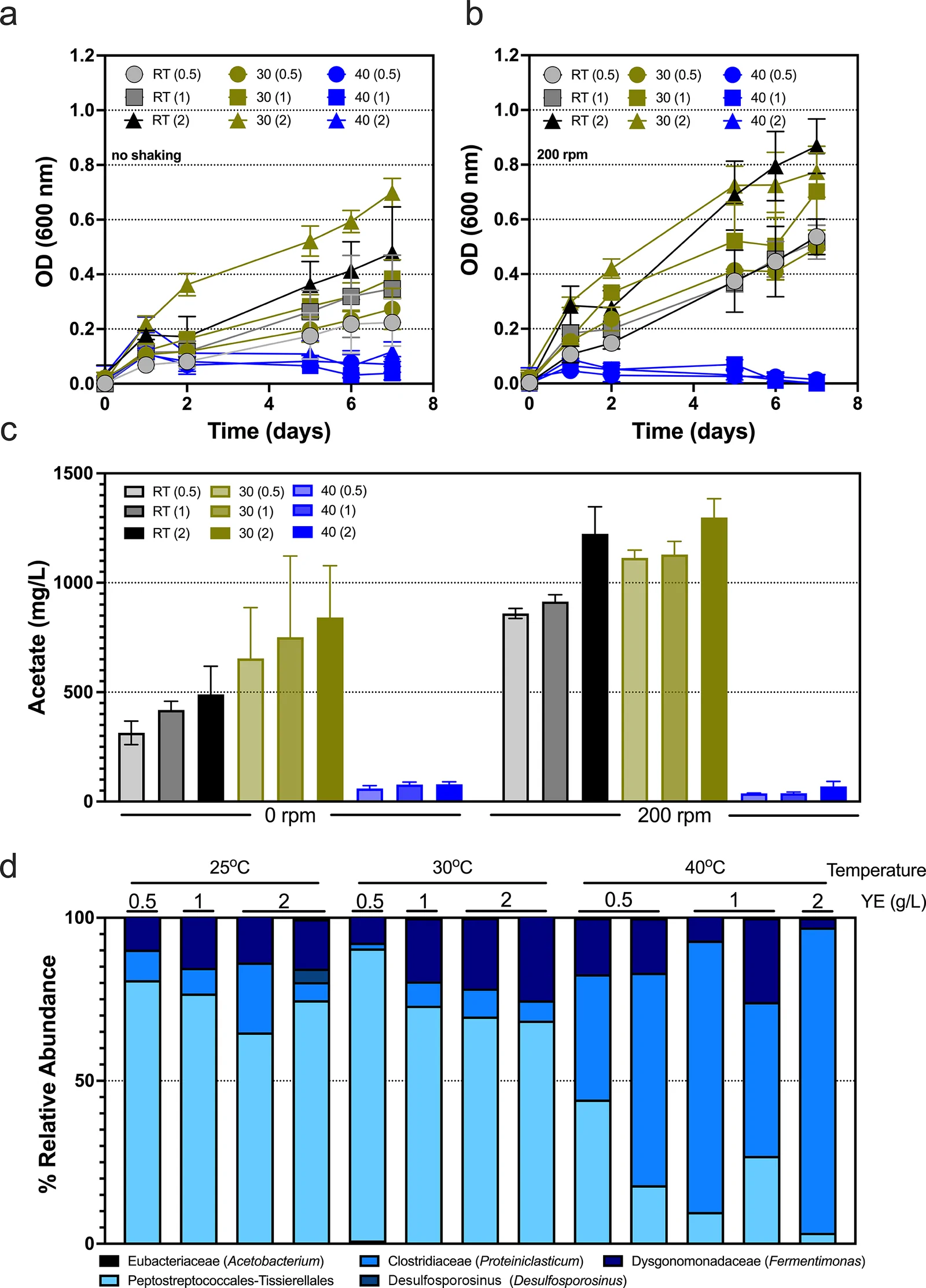
CSPW microbiome development and the effects of agitation, temperature, and yeast extract. (**a**) Growth curves of enrichment cultures incubated for 7 days at room temperature (RT) (black), 30°C (yellow), and 40°C (blue), with no agitation. (**b**) Growth curves of enrichment cultures with agitation (200 rpm). (**c**) Acetate concentrations at day 7 for incubations at room temperature (RT), 30°C, and 40°C, with addition of 0.5, 1, or 2 g/L yeast extract. (**d**) Relative abundance of microbial taxa determined via amplicon sequencing of the 16S rRNA gene.

To maximize growth rates and product titers, several variables were tested, including agitation, temperature, and yeast extract (YE) concentration. Experiments were executed with the CSPW microbiome over a 7-day period with and without agitation, and with varying temperatures (room temperature [~25°C], 30°C, and 40°C) and yeast extract (YE) (0.5, 1, and 2 g/L) (Fig. 2). Agitation had a positive effect on growth and acetate titers, as incubations that were agitated at 200 rpm grew faster, had higher final ODs, and produced more acetate than stagnant cultures (no shaking). Incubations at 30°C yielded the fastest growth and highest acetate titers, followed by 25°C, while incubations at 40°C exhibited the least amount of growth and produced small amounts of acetate (< 100 mg/L). Addition of yeast extract (YE) contributed to increased growth rates and acetate titers, with the largest increases occurring between 1 and 2 g/L YE.

Next, to understand what types of microorganisms were present in the enriched CSPW microbiome, how experimental parameters may affect the microbial community, and how the microbial community composition may relate to product formation, 16S rRNA gene amplicon sequencing was performed (Fig. 2). All samples were comprised of the following taxa (>1% relative abundance): Peptostreptococcales-Tissierellales, Clostridiaceae (*Proteiniclasticum*), Desulfobacteraceae (*Desulfosporosinus*), Dysgonomonadaceae (*Fermentimonas*), and Eubacteriaceae (*Acetobacterium*). Peptostreptococcales-Tissierellales was resolved to the order level and had the highest relative abundances at all yeast extract concentrations at 25°C and 30°C, with relative abundances ranging from 64.9% to 89.6% (Fig. 2). The next most abundant taxa were resolved to the genus level and included *Fermentimonas*, ranging from 7.6% to 25.3%, followed by *Proteiniclasticum* (1.7%–21.4%), *Desulfosporosinus* (0.05%–4.14%), and *Acetobacterium* (0.4%–1.0%). The taxonomic profile at 40°C was markedly different than 25°C and 30°C, with *Proteiniclasticum* having the highest representation (38.4%–93.7%), followed by Peptostreptococcales-Tissierellales (3.3%–43.9%), *Fermentimonas* (2.9%–25.8%), and *Acetobacterium* (0.11%–0.32%).

### Sodium bicarbonate is required for growth and product formation

**Fig 3:**
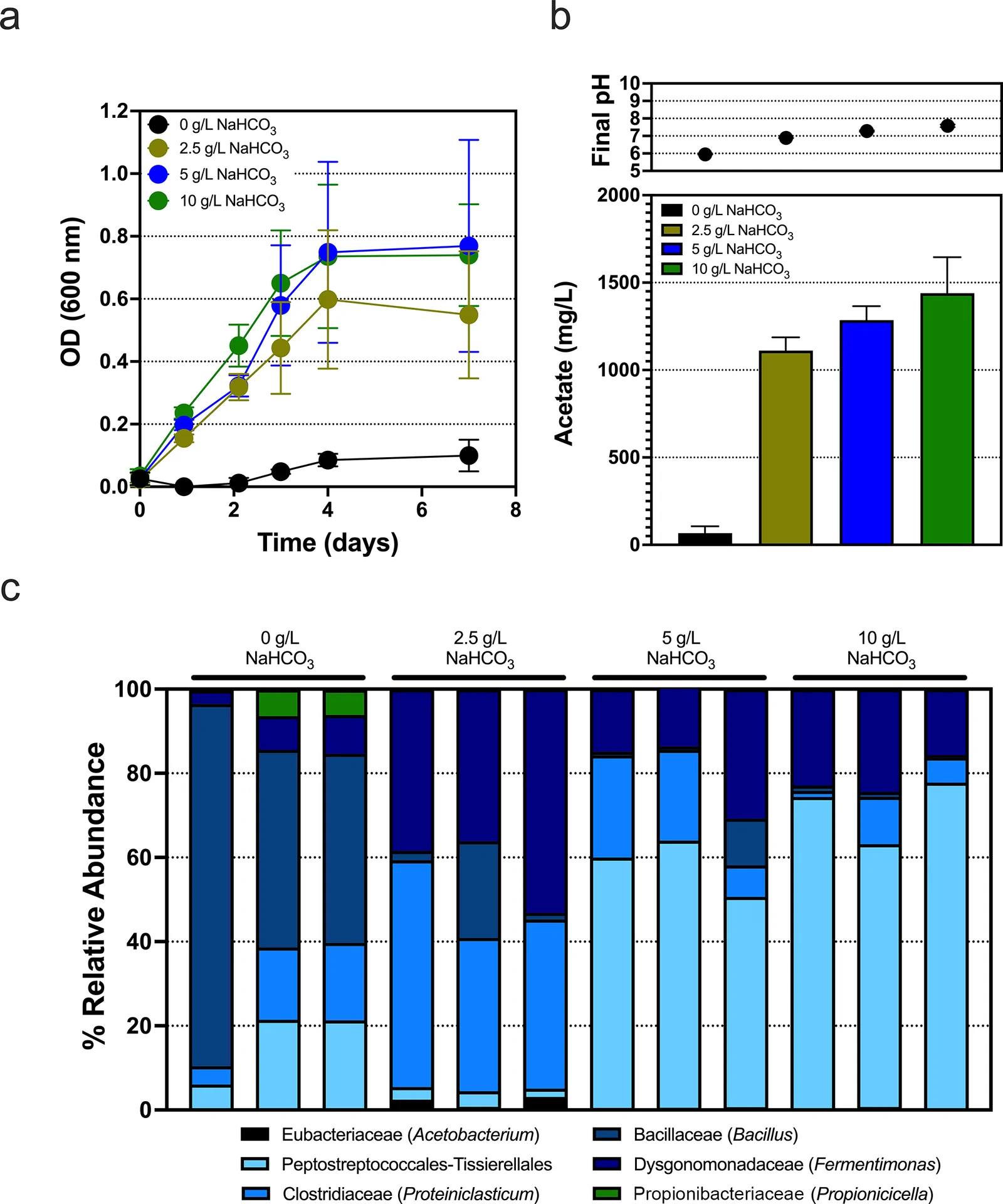
CSPW microbiome growth and acetate production is dependent on sodium bicarbonate. (**a**) Growth curves, (**b**) pH and acetate concentration at day 7, and (**c**) microbial community composition of samples.

Next, we examined a range of sodium bicarbonate (NaHCO_3_) concentrations (0, 2.5, 5, and 10 g/L) to determine its effect on growth and product formation. Initial pH values of the uninoculated medium ranged from 5.50 (0 g/L NaHCO_3_) to 7.24 (10 g/L NaHCO_3_). Cultures with no NaHCO_3_ exhibited minimal growth (OD_600_ = 0.1), produced small amounts of acetate (~41–164 mg/L), and had a final pH of 5.95 (Fig. 3a and b). With the addition of 2.5 g/L NaHCO_3_, growth increased to a final optical density (OD) of 0.549, produced ~10× more acetate (1.11 g/L), and had a final pH of 6.88. At 5 g/L NaHCO_3_, cultures reached a final OD of 0.769, produced 1.29 g/L acetate, and had a final pH of 7.28. Incubations with 10 g/L NaHCO_3_ grew to a final OD of 0.739, produced 1.44 mg/L acetate, and had a final pH of 7.58 (Fig. 3a and b). Specifically, higher acetate production corresponded to higher culture optical densities, with a range of optical densities from 0.4 to 1.2 producing over 1.0 g/L acetate (Fig. 3). Furthermore, the highest acetate titers and highest optical densities occurred in a pH range of 6.9–7.6. Overall, increased NaHCO_3_ led to increased pH, cellular growth, and acetate production, suggesting a role of NaHCO_3_ as a pH buffer and/or a source of inorganic carbon.

The relative abundance of the main taxa varied with NaHCO_3_ concentration. Peptostreptococcales-Tissierellales was the predominant taxon in enrichments containing 5 or 10 g/L NaHCO_3_, with relative abundances ranging from 50% to 77% (Fig. 3f). The next most abundant taxa were *Fermentimonas* (13.7%–30.8%) and *Proteiniclasticum* (5.9%–24.3%). Enrichments had markedly different community structures below 2.5 g/L NaHCO_3_. The 0 g/L NaHCO_3_ enrichments were predominantly *Bacillus* (45%–85%), followed by Peptostreptococcales-Tissierellales (5.8%–21%), *Proteiniclasticum* (4.4%–18%), *Fermentimonas* (3.1%–9.2%), and *Propionicicella* (0%–6.3%), while the 2.5 g/L enrichment was mainly *Fermentimonas* (40%–55%) and *Proteiniclasticum* (40%–60%), followed by *Bacillus*, Peptostreptococcales-Tissierellales, and *Acetobacterium*. These findings highlight two interesting points: (i) the Peptostreptococcales-Tissierellales is highly dependent on sodium bicarbonate, showing a large decrease in relative abundance from 5 g/L NaHCO_3_ (50%–60%) to 2.5 g/L NaHCO_3_ (2%–4%), and (ii) *Proteiniclasticum* and *Fermentimonas* were highly abundant at 2.5 g/L NaHCO_3_ with optical density and acetate titers decreasing slightly, suggesting a robust microbial community resilient to environmental perturbations.

### CSPW microbiome at larger volumes and longer duration

**Fig 4:**
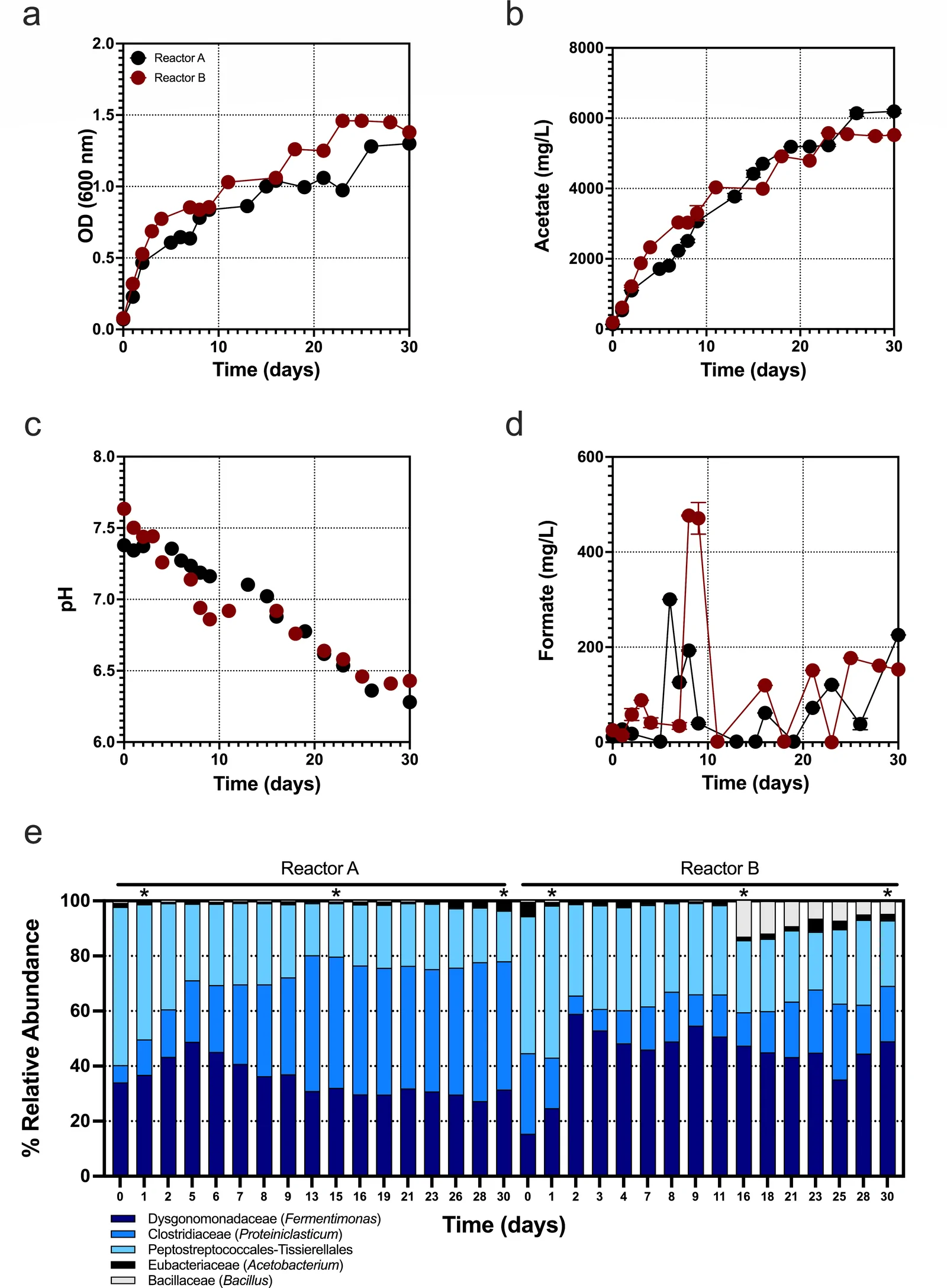
Larger-scale semi-batch reactor performance; 1 L reactors with 500 mL liquid volume were operated at 30°C with continuous stirring. Reactors were operated in semi-batch mode, with headspace gas replenished at each time point. (**a**) Optical density (600 nm), (**b**) acetate (mg/L), (**c**) pH, (**d**) formate (mg/L), and (**e**) microbial community relative abundance were monitored for 30 days. *Denotes samples extracted for metagenome sequencing.

To examine the CSPW microbiome carbon conversion at larger volumes (70), we scaled up batch reactors from 20 mL (10 mL working volume) to 1 L (500 mL working volume) (Fig. 4). Duplicate reactors were operational for 30 days in a semi-batch mode, with no additional pH control other than the initial sodium bicarbonate buffer. The gas headspace was flushed with H_2_:CO_2_ (80:20) at each time point to maintain anaerobicity. Microbial growth was fastest over the first few days and slowed for the duration of the experiment. The pH remained stable over the first 5 days (~7.3–7.5), but from day 5 to day 30, it slowly decreased to 6.3. Over the first 7 days, reactors produced 2.23 and 3.03 g/L of acetate, compared to 1.0–1.6 g/L of acetate for small batch reactors. After 30 days of operation, acetate titers reached 5.52 and 6.19 g/L. Acetate production rates were highest over the first 4 days of operation (0.582 g/L/day), slowed to an average of 0.366 g/L/day over the first 11 days, and had an average acetate production of 0.184 g/L/day over 30 days. Formate accumulated at various time points throughout, with the highest spikes between day 5 and day 10, and corresponded to slowing growth rates, and a stalling in acetate production. This phenomenon may be indicative of a switch from exponential growth strategies to the stationary phase and suggests that formate is a potential intermediate.

The taxonomy of the enriched CSPW microbiome was monitored over the course of the experiment and was comprised of *Fermentimonas*, *Proteiniclasticum*, Peptostreptococcales-Tissierellales, *Acetobacterium*, and *Bacillus* (Fig. 4). At the start of the experiment, Peptostreptococcales-Tissierellales was the most abundant (relative abundance ~60%), followed by *Fermentimonas* and *Proteiniclasticum. Fermentimonas* increased over the first few days (from ~15%–30% to ~45%–60%) and decreased over the remainder of the experiment to a final relative abundance of ~30%–45%. *Proteiniclasticum* increased over the course of the experiment and remained relatively stable from day 2 onward. *Fermentimonas* was slightly elevated in reactor 2 (~40%–60% relative abundance), compared to reactor 1 (~30%–50%). Conversely, the relative abundance of *Proteiniclasticum* was elevated in reactor 1 (~20%–50%) compared to reactor 2 (~5%–10%). Interestingly, *Bacillus* was present in both reactors (<1% relative abundance), but in reactor 2, *Bacillus* increased to 12% relative abundance, slowly decreasing to 4.7% at the end of the experiment (Fig. 4). Despite variations in relative abundances among reactors, no major differences were observed in changes in growth, pH, and acetate production for each reactor, suggesting potential metabolic overlap between microbial community members and corroborating previous findings of a robust and adaptable enriched microbiome.

### Hydrogen enhances growth and acetate production

**Fig 5:**
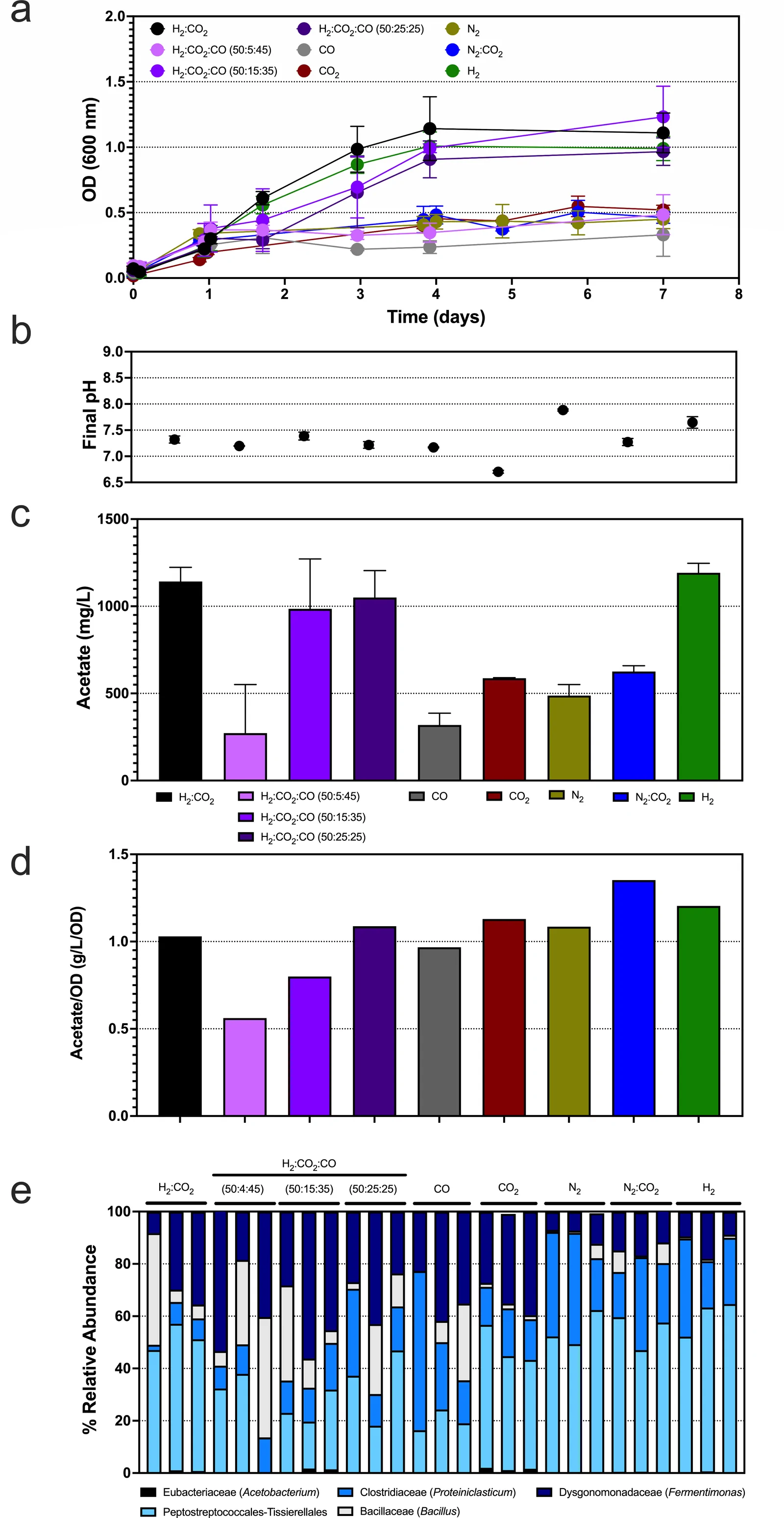
Growth and acetate production with a variety of pure and mixed anaerobic gases. Anaerobic batch experiments (10 mL working volume) were gassed with various pure and mixed gases and incubated at 30°C with shaking (200 rpm). (**a**) CSPW microbiome growth was monitored over 7 days. (**b**) The pH of filtered culture supernatant was measured on day 7. (**c**) Acetate titers were determined from filtered culture supernatants. (**d**) Acetate titers normalized to optical density. (**e**) 16S rRNA gene amplicon-based taxonomy.

Thus far, we have shown that the enriched microbiome exhibits the best growth and acetate production when supplied with 1–2 g/L YE and 10 g/L sodium bicarbonate incubated at 30°C with agitation (200 rpm). To examine the ability of using alternative gas substrates, growth of the CSPW microbiome and organic acid production was examined using various pure and mixed gases (Fig. 5). Growth was most robust with H_2_:CO_2_, H_2_, H_2_:CO_2_:CO (50:15:35), or H_2_:CO_2_:CO (50:25:25), reaching max optical densities of 1–1.25, followed by CO_2_, H_2_:CO_2_:CO (50:5:45), N_2_, N_2_:CO_2_, and CO, with optical densities ranging from 0.45 to 0.52 (Fig. 5a). Final pH values at the end of each experiment varied, depending on the supplied gas. For example, H_2_:CO_2_ enrichments had a final pH of 7.32, CO_2_ had a lower pH (6.70), and H_2_, N_2_, and N_2_:CO_2_ had a higher final pH (8.99, 9.20, and 8.26, respectively) (Fig. 5b). Acetate production was observed with all gases tested, with H_2_:CO_2_ producing the most (1.2 g/L), followed by H_2_ (1.19 g/L), H_2_:CO_2_:CO (50:25:25) (1.05 g/L), H_2_:CO_2_:CO (50:15:35) (0.99 g/L), N_2_:CO_2_ (0.63 g/L), CO_2_ (0.59 g/L), N_2_ (0.49 g/L), CO (0.32 g/L), and H_2_:CO_2_:CO (50:25:25) (0.27 g/L) (Fig. 5c). Depending on the supplied gas, the dominant taxa of the CSPW microbiome varied (Fig. 5d). Peptostreptococcales-Tissierellales had the highest relative abundances and were predominant in samples containing H_2_:CO_2_, CO_2_, N_2_, N_2_:CO_2_, or H_2_. Conversely, experiments containing any amount of CO had markedly different community structures, including a higher relative proportion of Bacillaceae and lower relative abundance of Peptostreptococcales-Tissierellales. In all cases, irrespective of gas composition, the main taxa were still present, albeit at varying relative abundances. Overall, hydrogen-containing gases yielded the highest growth and acetate titers, even in the presence of carbon monoxide, suggesting that syngas could be utilized as a gaseous feedstock.

### Enriched CSPW microbiome characterization and quality assessment of metagenome-assembled genomes

**Fig 6:**
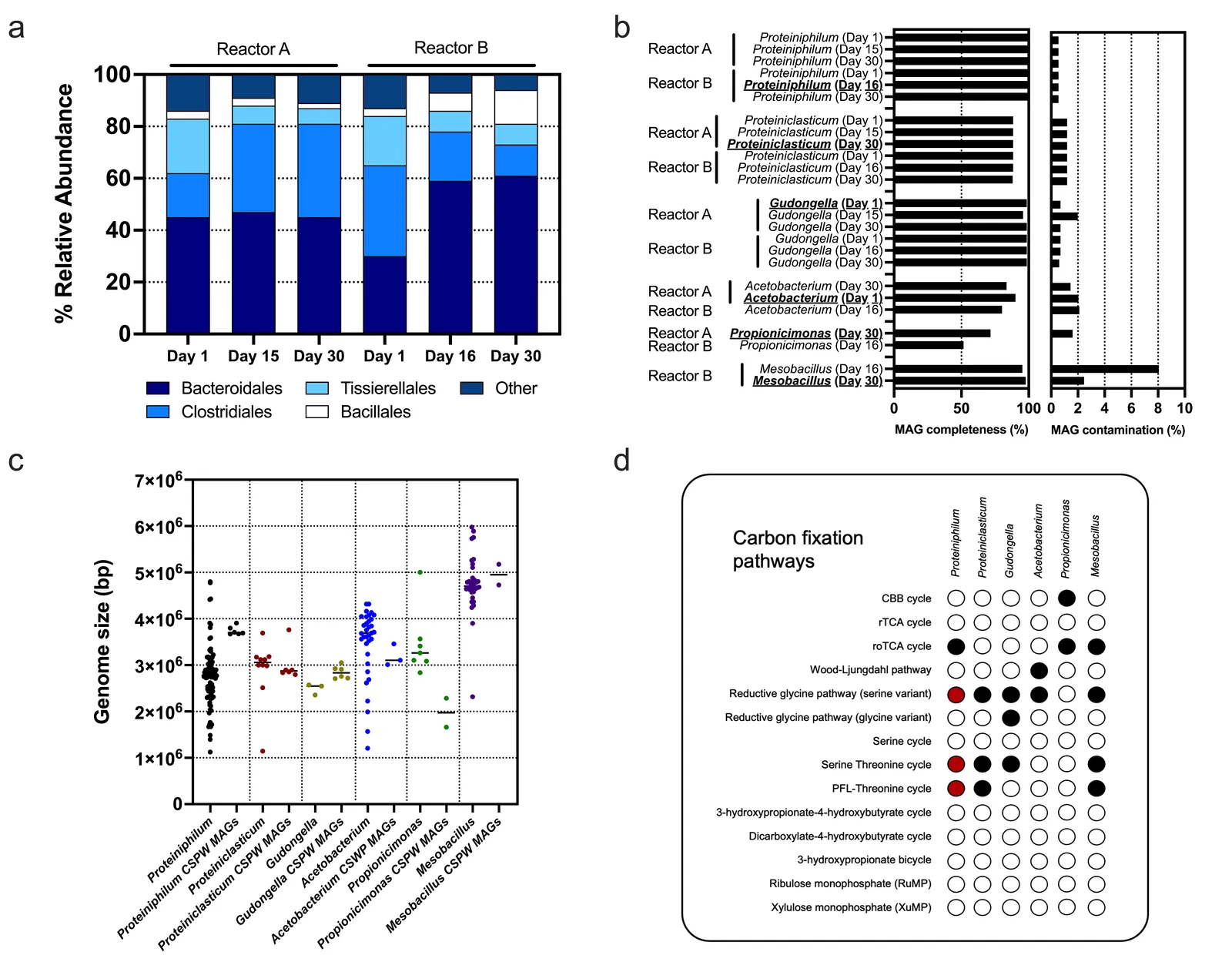
Taxonomic characterization of metagenomes and quality assessment of MAGs from the CSPW microbiome enrichment. (**a**) Taxonomy of trimmed and filtered paired-end metagenome reads (as determined by Kaiju). (**b**) Completeness and contamination of MAGs (as determined by CheckM). (**c**) Comparison of genome sizes for CSPW MAGs and genomes/MAGs from the same genus. (**d**) Overview of carbon fixation pathways present in CSPW MAGs. Black circle denotes full pathway, white circle denotes no pathway or more than one missing enzyme, and red circle denotes only one enzyme missing.

Metagenome sequencing was performed on duplicate 1 L H_2_:CO_2_ reactors on days 1, 15 (or 16), and 30 (Fig. 6). Taxonomic identification of unassembled metagenome reads revealed similar relative abundances to what was observed with 16S rRNA gene amplicon sequencing (Fig. 6a). The total length of assembled metagenome reads ranged from ~9.6 to 19.3 Mbp, with the total number of contigs ranging from 478 to 2,139. Contig N50 ranged from 18,539 to 258,324 bp (Table S3). MAGs were generated via assembly and binning of metagenome reads. Across all samples, MAGs were classified into six different bacterial genera, including four Firmicutes (*Gudongella*, *Proteiniclasticum*, *Acetobacterium*, and *Mesobacillus*), one Bacteroidota (*Proteiniphilum*), and one Actinobacteriota (*Propionicimonas*) (Fig. 6b; Table S4). Based on the presence of single copy marker genes, the MAGs were estimated to be between ~71% (*Propionicimonas*) to 100% complete (*Proteiniclasticum*), with most MAGs greater than 88% complete and containing <2% contamination (Fig. 6b; Table S4). Approximately 98% of the paired metagenome reads for each sample mapped to the MAGs generated from that sample, signifying adequate representation of the metagenome in the MAGs. For example, in one sample, 42.30% of paired reads mapped to the *Proteiniphilum* MAG, 35.45% mapped to the *Proteiniclasticum* MAG, 16.18% mapped to the *Gudongella* MAG, 3.09% mapped to the *Acetobacterium* MAG, and 1.57% mapped to the *Propionicimonas* MAG. Except for *Propionicimonas*, all MAG sizes fell within the range of genome sizes for members of the same genus (Fig. 6c). It should be noted that 16S rRNA gene amplicon sequencing revealed the presence of *Fermentimonas* and Peptostreptococcales-Tissierellales; however, we found no evidence of *Fermentimonas* in our metagenome data and instead found *Proteiniphilum*, which is closely related to *Fermentimonas*. Likewise, based on taxonomic characterization and similarity searches of amino acid sequences, Peptostreptococcales-Tissierellales most likely corresponds to *Gudongella*, which is part of the family *Tissierellaceae*. Since MAGs of the same genus were present throughout the experiment, whole genome comparisons were performed (average nucleotide identity) and revealed high similarity (>99%) across samples, suggesting that, for example, the *Proteiniphilum* sp. MAG was the same organism recovered at each time point (Table S4).

### Genome-resolved metabolisms of autotrophic pathways

To elucidate how carbon is being utilized by the CSPW microbiome for growth and acetate production, we examined metagenome-assembled genomes for all known autotrophic pathways ((71), and references therein). Only the highest quality MAG from each taxon was used to elucidate autotrophic pathways (Fig. 6; Table S4). A survey of the CSPW MAGs revealed six potentially operative pathways, including four known natural pathways—the reductive pentose phosphate pathway (CBB cycle), the reversed oxidative TCA cycle, the reductive Acetyl-CoA/WLP, and the reductive glycine pathway (rGlyP)—and two proposed synthetic pathways—the serine threonine cycle (SerThrCyc) and the pyruvate formate-lyase (PFL)-threonine cycle (PFL-ThrCyc) (Fig. 6d).

#### The reductive pentose phosphate pathway (CBB cycle)

**Fig 7:**
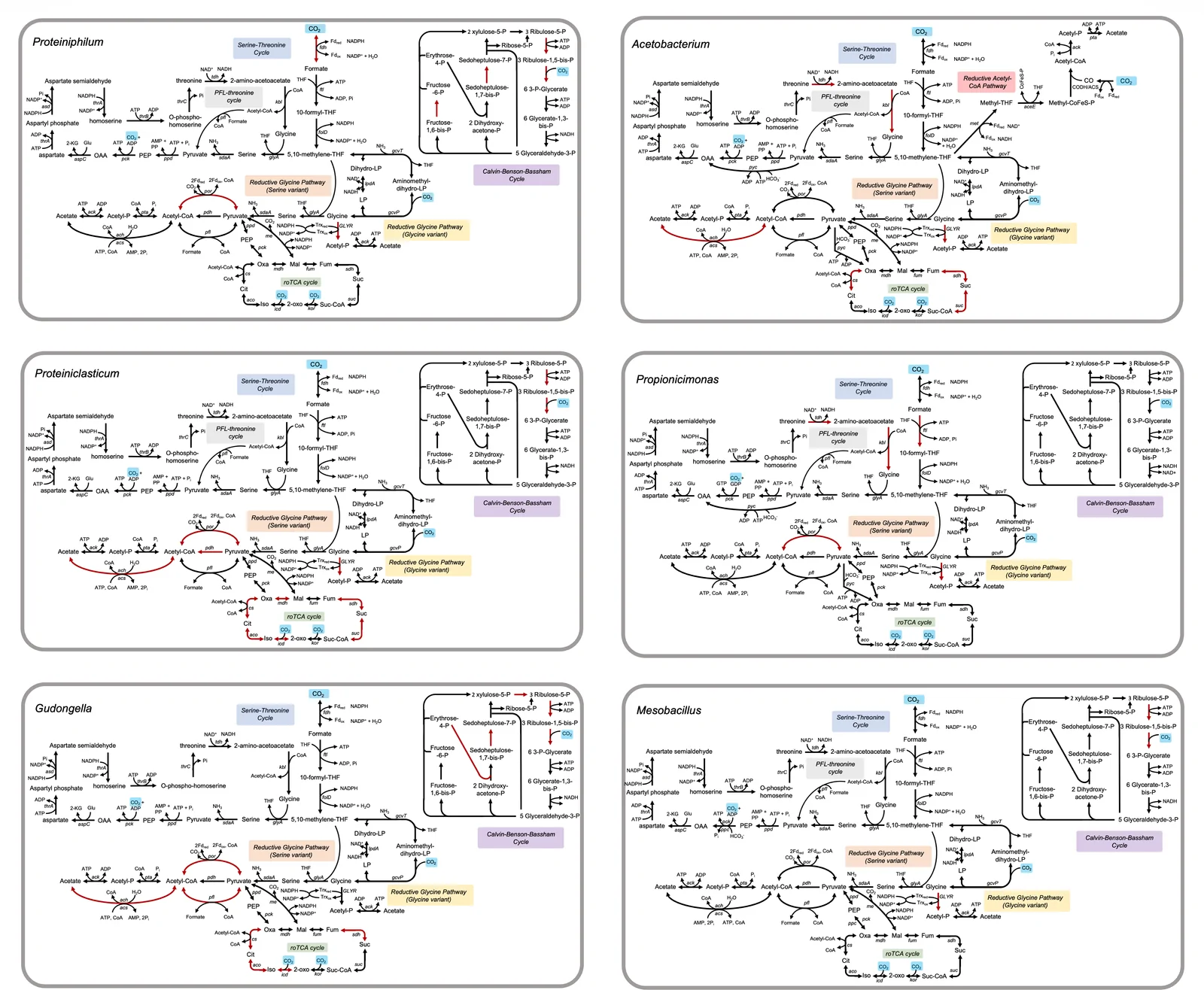
Overview of carbon fixation pathways present in CSPW MAGs. Black line denotes gene presence, red line denotes gene absence. Pathway details can be found in Tables S5 to S9.

The CBB cycle converts atmospheric CO_2_ into organic matter, driven by ribulose-1,5-bisphosphate carboxylase/oxygenase (RubisCO), and is responsible for most of the carbon fixation in the biosphere. The two key enzymes, RubisCO and phosphoribulokinase (PRK), were found in the *Propionicimonas* MAG only (Fig. 7; Table S5). The *Propionicimonas* MAG neither encoded sedoheptulose-1,7-bisphosphate aldolase (SBA) nor sedoheptulose-1,7-bisphosphatase (SBP). Thus, if the CBB pathway is operable, either the fructose-1,6-bisphosphate aldolase (FBA) and fructose-1,6-bisphosphatase (FBP) have promiscuous activity to catalyze these reactions (72, 73) or a transaldolase variant is operative (74).

#### The reductive tricarboxylic acid cycle and reversed oxidative TCA cycle

The TCA cycle can operate in reverse for autotrophic metabolism (16, 17), using either ATP-citrate lyase (rTCA) or citrate synthase (roTCA) to overcome reactions that are irreversible under physiological conditions (75). The roTCA cycle requires 2-oxoglutarate synthase (ferredoxin-dependent) and high levels of CO_2_ (75) and has been observed in a variety of microorganisms, including *Salinilacustristhrix flava* (76), *Hippea maritima* (75), *Geobacter sulfurreducens* (77), *Desulfurella acetivorans* (16), *Thermosulfidibacter takaii* (17), and *Deferribacter autotrophicus* (78). ATP-citrate lyase was not encoded in any CSPW MAG, ruling out the rTCA cycle, but the *Proteiniphilum, Propionicimonas,* and *Mesobacillus* MAGs encoded for citrate synthase and 2-oxoglutarate:ferredoxin oxidoreductase, suggesting that the roTCA cycle may be operative (Fig. 7; Fig. S3; Table S6).

#### The reductive acetyl-CoA/Wood-Ljungdahl pathway

The WLP is an ancient anaerobic autotrophic pathway employed by acetogens and is the most efficient anaerobic CO_2_ fixation pathway (79). The *Acetobacterium* MAG likely utilizes this pathway, as it was the only MAG to encode 5,10-methylene-THF reductase (*met*), 5-methyl-THF methyltransferase (*aceE*), and the carbon monoxide dehydrogenase/acetyl-CoA synthase (CODH/ACS) (Fig. 7; Table S7). The WLP is well characterized in *Acetobacterium woodii* (80), and BLASTp comparison with the CSPW *Acetobacterium* MAG (Table S7) revealed a complete WLP. In *Acetobacterium woodii,* the WLP is organized into three distinct clusters, with one encoding formate dehydrogenase (FDH) and accessory proteins, one encoding the methyl branch, and one encoding the carbonyl branch (80). The CSPW *Acetobacterium* MAG contained a highly conserved methyl and carbonyl branch; however, the FDH cluster was markedly different from the *A. woodii* FDH. These findings are in line with previous research showing a divergent formate dehydrogenase gene cluster among strains of the *Acetobacterium* genus (81).

#### The reductive glycine pathway

The *rGlyP* is a pathway found in amino-acid degrading (82) and purine-utilizing (83) *Clostridia* spp. and more recently *Desulfovibrio desulfuricans* (7) and *Vibrio natriegens* (84). The rGlyP can be separated into three modules: the glycine biosynthesis module; the oxygen-tolerant glycine-to-serine conversion module and the oxygen-sensitive glycine-to-acetyl-phosphate module; and the pyruvate synthesis module (85).

In the core module (i.e., glycine biosynthesis module), formate and CO_2_ are converted to glycine. All MAGs encode the core module, except for *Proteiniphilum* and *Propionicimonas,* which are missing formate dehydrogenase and formate-tetrahydrofolate ligase, respectively (Fig. 7; Table S8). The glycine-to-serine module (i.e., serine variant or serine deaminase pathway) glycine is converted to serine. The serine variant employs serine hydroxymethyltransferase for conversion of glycine to serine, which was encoded by all MAGs. The pyruvate synthesis module corresponds to the serine variant of the rGlyP, where serine is converted to pyruvate (via serine deaminase), and all MAGs encoded for this module. Pyruvate then feeds into central carbon metabolism and can be converted to acetyl-CoA by pyruvate dehydrogenase.

The glycine-to-acetyl-phosphate module requires the activity of glycine reductase and has been termed the glycine synthase-reductase pathway (or glycine reductase variant) (86). Glycine reductase is oxygen-sensitive and selenium-dependent, and only the *Gudongella* MAG contained this enzyme, which encodes thioredoxin reductase (*trxA/trxB*) and glycine reductase (*grdA, grdB, grdC,* and *grdD*) in a single operon (Table S8). Acetyl-phosphate can be converted to acetate via acetate kinase, generating 1 ATP in the process.

The genomes of many heterotrophic and acetogenic microorganisms encode the rGlyP (87), and the serine variant was much more prevalent than the glycine-synthase variant (7). We find this to hold true in our system, with most MAGs encoding for the serine variant, and only the *Gudongella* MAG encoding for the serine and glycine reductase variant.

#### The proposed synthetic serine-threonine cycle (SerThrCyc) and pyruvate formate-lyase (PFL)-threonine cycle (PFL-ThrCyc)

The serine cycle is a natural pathway mainly employed by methylotrophs (13, 88, 89) and is relatively inefficient, supporting moderate growth yields (90). While no MAG encoded for the full serine pathway, the SerThrCyc, which is a proposed synthetic variant of the serine pathway (91–94), was encoded by the *Proteiniclasticum, Gudongella,* and *Mesobacillus* MAGs (Fig. 7; Table S9). The SerThrCyc utilizes L-serine ammonia-lyase (EC 4.3.1.17) for the conversion of serine to pyruvate + NH_3_ instead of serine-glyoxylate transaminase (EC 2.6.1.45), which converts serine and glyoxylate into 3-hydroxypyruvate and glycine. Furthermore, unlike the serine cycle, which utilizes malyl-CoA synthase and malyl-CoA lyase, the SerThrCyc employs enzymes of threonine synthesis/degradation, with 2-amino-3-ketobutyrate CoA ligase (EC 2.3.1.29) producing acetyl-CoA and glycine from 2-amino-acetoacetate in the final step of the cycle. Recently, the SerThrCyc was implemented in *E. coli* for formatrophic growth (92).

The PFL-threonine cycle (PFL-ThrCyc) is a proposed variation of the synthetic SerThrCyc, whereby pyruvate formate-lyase (PFL) is used as the formate-fixing reaction instead of formate-tetrahydrofolate ligase (91). All MAGs encoded for PFL except for the *Gudongella* MAG, although only the *Proteiniclasticum* and *Mesobacillus* MAGs encoded for all remaining steps of the pathway. Therefore, the PFL-threonine cycle represents another possible autotrophic route within the CSPW microbiome.

## DISCUSSION

Coal seams harbor a diverse network of microorganisms with a wide variety of metabolisms (20, 22, 23, 29, 95), and this diversity is observed across various coal basins and within samples from the same basin (25). Previously, metagenomic data from Appalachian Basin CSPW revealed a predominance of Methanomicrobiales in most wells sampled (25). Samples from this study were similar, in that two of the samples had the highest relative abundances of Methanomicrobiales (NV57 [36%] and NV73 [19%]), and one was predominantly Eubacteriales (NV65 [77%]). Amplicon sequencing revealed numerous taxa from our samples correspond to taxa found in coal seams from other basins (Table S10). Interestingly, sample NV65 contained ~4% relative abundance of known methanogens; however, no methanogens were detected in our enriched microbiome. Under our experimental conditions, it is possible that inadvertent oxygen exposure inhibited methanogen growth, and/or the medium constituents favored growth of the six bacterial taxa we observed. Geochemical characterization of CSPW revealed similar characteristics to previously observed geochemistry of Appalachian Basin CSPW samples from a different region (25). The Appalachian Basin samples had much higher TDS and major cations/anions compared to samples from geographically distinct coal basins (Table S1). Therefore, areas of the San Juan Basin are associated with high TDS (14,000–42,000 mg/L) (96). Taken together, coal seams are heterogeneous environments with varied microbiology and geochemistry, and samples from the Appalachian Basin have distinct geochemical and microbiological signatures compared to other basins.

The enriched CSPW microbiome was able to fix carbon dioxide, in the form of dissolved CO_2_ and/or bicarbonate, for growth and acetate production. Multiple lines of evidence suggest that gaseous H_2_ and CO_2_ were being consumed by the CSPW microbiome. In many cases, most notably in the larger-scale (1 L) reactors, a strong negative pressure was observed over the course of the experiment, suggesting utilization of gaseous components. Second, optical densities and acetate production were greatest when hydrogen gas was supplied (H_2_, H_2_:CO_2_, and H_2_:CO_2_:CO). This implies that CO_2_ fixation (via gaseous CO_2_ or dissolved bicarbonate) required H_2_ that would act as a reductant, replenishing oxidized NAD(P)+ and ferredoxin pools, potentially through the activity of hydrogenases, which were encoded in all MAGs except *Mesobacillus*.

The mole fraction of inorganic carbon is predominantly bicarbonate (HCO_3_^-^) between pH 7.5 and 9 (97). This holds true for dissolved inorganic carbon (DIC) from deep subsurface hydrothermal fluids with neutral to slightly alkaline pH (98). In most incubations tested here, final pH values ranged from 7 to 8, and as such, HCO_3_^-^ not only acts as a buffer but also serves as a carbon fixation channel. Many alternative entry points for bicarbonate exist, including phosphoenolpyruvate carboxylase (encoded by the *Mesobacillus* MAG), acetyl-CoA carboxylase (*Proteiniclasticum,* and *Mesobacillus*), carbonic anhydrase (encoded by all MAGs), carbamoyl-phosphate synthase (encoded by all MAGs except *Gudongella*), and pyruvate carboxylase (feeds into TCA cycle) (encoded by *Acetobacterium* and *Propionicimonas*) (Table S11). In the case of carbonic anhydrase, direct conversion of bicarbonate into CO_2_ would supply gaseous CO_2_ to all autotrophic pathways being expressed.

Yeast extract contains essential vitamins, amino acids, nitrogen, and carbon and is often used as a supplement for growth medium (99–101). As a nitrogen source, YE stimulates cell growth and hydrogen production by *Rhodobacter sphaeroides* (102) and outperforms other carbon sources for H_2_ production in *Clostridium acetobutylicum* (99). Under our experimental conditions, a minimal amount of YE was required for growth and acetate production, and carbon balances revealed that more carbon was contained in acetate and biomass compared to YE (Table S12), suggesting that assimilated carbon mainly originated from gaseous CO_2_ and/or HCO_3_^-^. Initial growth rates and final OD values showed greater enhancement with increasing YE concentrations compared with acetate production, suggesting that YE contributes more to the CSPW microbiome growth and, to less of an extent, acetate production. We hypothesize that yeast extract supplies the needed amino acids (AA) to the CSPW microbiome, as many MAGs did not encode specific AA biosynthesis pathways (Table S13). The most common AA auxotrophy was methionine and lysine, followed by arginine, asparagine, isoleucine, and tyrosine. Furthermore, the presence of enzymes involved in Stickland fermentation suggests that this metabolism may help support growth on amino acids (Table S13). While higher YE concentrations resulted in increased growth and acetate titers, only a minimal amount of YE was required, suggesting that carbon conversion is possible in nutrient-poor conditions (e.g., coal seams), but YE or alternative nutrient amendments (e.g., corn steep liquor, malt extract, or vegetable extract (103)) should be considered for development of the enriched CSPW microbiome as a biocatalyst for carbon conversion technologies.

Carbon conversion within the CSPW microbiome may be complex and nuanced, with multiple microorganisms contributing to carbon fixation via multiple pathways. It is also possible that each microorganism utilizes a single autotrophic pathway or that a single microorganism is responsible for the majority of carbon conversion, while the others utilize metabolic intermediates for growth. Many of the MAGs encoded for multiple autotrophic pathways, including *Proteiniclasticum* (rGlyP, SerThrCyc, and PFL-ThrCyc), *Gudongella* (rGlyP and SerThrCyc), *Acetobacterium* (WLP and rGlyP), *Propionicimonas* (CBB and roTCA), and *Mesobacillus* (rGlyP, SerThrCyc, PFL-ThrCyc, and roTCA). It is unknown why multiple autotrophic pathways are present and whether they are operative, but recent work has revealed, in some cases, that these pathways may functionally cooperate to enhance CO_2_ consumption and cellular growth rates, as was observed in *Clostridium drakei* using the WLP and the rGlyP (86). Conversely, they may have distinct yet complementary roles for maintaining high carbon reaction rates in dynamic environments, as was proposed in the *Riftia pachyptila* chemoautotrophic symbiont “*Candidatus* Endoriftia Persephone” using the CBB cycle and rTCA cycle (104). In *Ammonifex degensii,* the CBB cycle and the WLP operate at low and high H_2_ partial pressure, respectively. Overall, to date, three autotrophic pathway combinations have been observed, including the CBB/WLP, the CBB/rTCA, and the rGlyP/WLP. Our data suggest that other combinations are possible, such as rGlyP/SerThrCyc/PFL-ThrCyc (*Proteiniclasticum*), rGlyP/SerThrCyc (*Gudongella*), CBB/roTCA (*Propionicimonas*), and rGlyP/SerThrCyc/PFL-ThrCyc/roTCA (*Mesobacillus*) (Table S14).

Of the six potentially operative autotrophic pathways, the reductive acetyl-CoA pathway (WLP) and the reductive glycine pathway (rGlyP) are the most favorable since they are both linear, contain the least number of reactions, utilize the least amount of resources (ATP, NAD(P)H), and are most energetically efficient (2). Conversely, the SerThrCyc and the PFL-ThrCyc contain the most reactions, require much more ATP, and have only been proposed as synthetic variants of the serine cycle (91). Likewise, the roTCA cycle, while promising, requires a high percentage of total expressed protein to be citrate synthase (75).

Based on this hypothesis, the main contributors of carbon reactions within the CSPW microbiome encode the rGlyP and/or the WLP. The most likely candidates are the *Gudongella* MAG, which encoded for the rGlyP (serine and glycine variant) and *Acetobacterium,* which encoded for the WLP and the rGlyP (serine variant). The *Proteiniphilum, Proteiniclasticum,* and *Mesobacillus* MAGs also encoded for the rGlyP (serine variant), but the *Proteiniphilum* MAG was missing formate dehydrogenase and cannot convert CO_2_ into formate. Although under conditions where formate was present, as was observed in larger-scale reactors (Fig. 4), *Proteiniphilum* would be able to utilize extracellular formate and produce acetate using the rGlyP. The production and reutilization of formate also suggest the reductive activity of formate dehydrogenase, as was observed in *Desulfovibrio desulfuricans* grown under autotrophic conditions (7), and/or the conversion of pyruvate to acetyl-CoA and formate via pyruvate formate-lyase.

### Future development and biotechnological aspects of the CSPW microbiome

Based on the findings of this study, we currently know the microorganisms present and the pathways that are potentially used to fix carbon, but we do not know which individual microorganisms are active and what pathways are active during this process. Metagenomics and genome-resolved metabolisms alone cannot yield this information. While we hypothesize that the major carbon inputs are gaseous CO_2_ and/or bicarbonate, with minor contributions from yeast extract (Fig. S4), to understand the flow of carbon through individual microorganisms and pathway activity, future experiments will center around isotopically labeled carbon feedstocks and/or metatranscriptomics. We could then decipher if pathways are differentially expressed under various growth conditions (e.g., N_2_ vs N_2_CO_2_ vs H_2_ vs H_2_CO_2_), if pathway activity changes during growth phase (e.g., exponential vs stationary phase), and what source of carbon is being utilized (e.g., gaseous CO_2_ vs bicarbonate).

Microbial gas fermentation has been well established for many isolate microorganisms, with the ability to naturally produce a variety of products, including acetate, ethanol, butanol, and 2,3-butanediol. Our CSPW enrichment routinely produced 1–1.5 g/L acetate, while semi-batch (1 L) reactors produced > 6 g/L acetate. For comparison, during autotrophic growth on H_2_:CO_2_ (80:20), *Eubacterium limosum* produced 1.23 g/L acetate, and *Clostridium ragsdalei* produced 1.99 g/L ethanol (105, 106). Our current batch mode operation allows for semi-high throughput to test numerous variables but is not ideal for scale-up. Industrial fermentation relies on alternative reactor configurations and flow-through systems to maximize productivity and efficiency (107, 108). For example, higher titers have been observed in specialized engineered reactors, with *Acetobacterium woodii* producing 44–59 g/L acetate (109, 110). Moving toward higher bioindustrial manufacturing readiness levels will require testing at larger volumes with constant exchange of gas and medium, and examination of feeding rates and pH control. Within this framework, to enhance economic viability, one must consider the carbon produced in the manufacturing of chemical inputs for microbial growth (e.g., yeast extract) and carbon produced/consumed in its conversion to acetate (111).

### Conclusion

In summary, we have provided geochemical and microbial characterization of CSPW from the Appalachian Basin, and through selective enrichment, we identified a unique microbial assemblage capable of growth and acetate production with carbon dioxide/bicarbonate. Multiple autotrophic pathways were identified from metagenome-assembled genomes, revealing the potential operation of the reductive glycine pathway, the reductive acetyl-CoA pathway, the reverse oxidative TCA cycle, the Calvin-Benson-Bassham cycle, the serine-threonine cycle, and/or the PFL-threonine cycle. Interestingly, many microorganisms encoded multiple autotrophic pathways, suggesting adaptable carbon conversion strategies. Overall, our findings shed light on potential autotrophic mechanisms within coal seams and provide a framework for the development of biological carbon conversion technologies.

## Data Availability

Metagenome reads from the original coal seam produced water samples (NV57, NV65, and NV73) and laboratory enrichment cultures were deposited in the NCBI database under the accession number PRJNA1328686.

## SUPPLEMENTAL MATERIAL

Supplemental figures aem.00624-26-s0001.docx Fig. S1 to S4.

Supplemental tables aem.00624-26-s0002.xlsx Tables S1 to S14.

ASM does not own the copyrights to Supplemental Material that may be linked to, or accessed through, an article. The authors have granted ASM a non-exclusive, world-wide license to publish the Supplemental Material files. Please contact the corresponding author directly for reuse.
