# Supplementary material for: Uncovering potential autotrophs in coal seams: characterization, enrichment, and genome-resolved metabolisms of a subsurface coal seam microbiome
Source: Appl Environ Microbiol. 2026 Sep 24;92(10):e00624-26. doi: 10.1128/aem.00624-26 (PMC13688921; doi:10.1128/aem.00624-26)
Supplement: Supplementary file 1 [file aem.00624-26-s0001.docx]

**Uncovering Carbon Fixation in Coal Seams: Characterization, Enrichment, and Genome-Resolved Metabolisms of a Subsurface Coal Seam Microbiome**

Daniel E. Ross^1,2^; Samuel Flett^1,2^; Djuna Gulliver^1*^

^1^National Energy Technology Laboratory, 626 Cochran Mill Road, Pittsburgh, PA 15236, USA

^2^NETL Support Contractor, 626 Cochran Mill Road, Pittsburgh, PA 15236, USA

*Corresponding author: djuna.gulliver@netl.doe.gov

**Running title:** CO_2_ conversion by a coal seam produced water microbiome.

**Competing interests:** The authors declare no competing financial interest.

**Supplemental Figures.**

Figure S1. Growth profiles of CSPW (1 mL) inoculated into various media (9 mL) under anaerobic conditions (H_2_:CO_2_, 80:20).

Figure S2. Maximum likelihood tree of MAGs from 1L enrichment cultures.

Figure S3. roTCA cycle.

Figure S4. Overview of carbon flow within the coal seam produced water microbiome. Most of the carbon utilized for biomass and acetate production originates from gaseous CO2 and/or sodium bicarbonate, while a small portion may come from yeast extract. The pathways encoded in each MAG are represented by small circles. Boxes with amino acid abbreviations denote potential amino acid utilization, including Stickland reactions.

**Figure S1. Growth profiles of CSPW (1 mL) inoculated into various media (9 mL) under anaerobic conditions (H_2_:CO_2_, 80:20).**

**Figure S2. Maximum likelihood tree of MAGs from 1L enrichment cultures.** The SpeciesTree (v2.2.0) application in Kbase was used to construct trees based upon 49 core/universal COG gene families. Samples were taken for metagenome sequencing on Day 1, 15/16, and 30.

**a.**

**b.**

**Figure S3. roTCA cycle**. A. Diagram of gene presence (black arrow) and absence (red arrow) across all CSPW MAGs. B. Protein sequence of citrate synthase aligned to the best BlastP hit. Highlighted amino acids represent conserved active site residues.

**Figure S4. Overview of carbon flow within the coal seam produced water microbiome.** Most of the carbon utilized for biomass and acetate production originates from gaseous CO2 and/or sodium bicarbonate, while a small portion may come from yeast extract. The pathways encoded in each MAG are represented by small circles. Boxes with amino acid abbreviations denote potential amino acid utilization, including Stickland reactions.
